# *N*′-[(2-Methoxynaphthalen-1-yl)methyl­idene]-4-methyl­benzohydrazide

**DOI:** 10.1107/S1600536811044291

**Published:** 2011-10-29

**Authors:** Xu-Feng Meng, Dong-Yue Wang, Jing-Jun Ma

**Affiliations:** a Hebei Key Laboratory of Bioinorganic Chemistry, College of Sciences, Agricultural University of Hebei, Baoding 071001, People’s Republic of China

## Abstract

In the title compound, C_20_H_18_N_2_O_2_, the mean planes of the naphthyl system and the benzene ring form a dihedral angle of 88.48 (10)°. In the crystal, N—H⋯O hydrogen bonds link the mol­ecules into *C*(4) chains, which propagate along the *b*-axis direction.

## Related literature

For the biological activity of benzohydrazide compounds, see: El-Sayed *et al.* (2011 ▶); Horiuchi *et al.* (2009 ▶). For coordination compounds of benzohydrazide compounds, see: El-Dissouky *et al.* (2010 ▶); Zhang *et al.* (2010 ▶). For standard bond lengths, see: Allen *et al.* (1987 ▶). For the crystal structures of similar compounds, see: Suleiman Gwaram *et al.* (2010 ▶); Liu *et al.* (2011 ▶); Zhou *et al.* (2011 ▶).
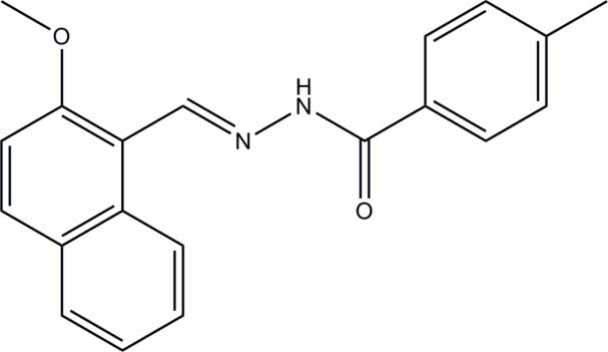


## Experimental

### 

#### Crystal data

C_20_H_18_N_2_O_2_
*M* *_r_* = 318.36
Orthorhombic, 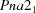

*a* = 26.738 (3) Å
*b* = 4.893 (2) Å
*c* = 12.735 (2) Å
*V* = 1666.1 (8) Å^3^
*Z* = 4
Mo *K*α radiation
μ = 0.08 mm^−1^
*T* = 298 K
0.20 × 0.20 × 0.18 mm

#### Data collection

Bruker SMART 1K CCD diffractometer
Absorption correction: multi-scan (*SADABS*; Sheldrick, 1996 ▶) *T*_min_ = 0.984, *T*_max_ = 0.985
12400 measured reflections
3622 independent reflections
2579 reflections with *I* > 2σ(*I*)
*R*_int_ = 0.047

#### Refinement

*R*[*F* ^2^ > 2σ(*F* ^2^)] = 0.043
*wR*(*F* ^2^) = 0.100
*S* = 1.03
3622 reflections
222 parameters
2 restraints
H atoms treated by a mixture of independent and constrained refinement
Δρ_max_ = 0.17 e Å^−3^
Δρ_min_ = −0.15 e Å^−3^

### 

Data collection: *SMART* (Bruker, 2007 ▶); cell refinement: *SAINT* (Bruker, 2007 ▶); data reduction: *SAINT*; program(s) used to solve structure: *SHELXS97* (Sheldrick, 2008 ▶); program(s) used to refine structure: *SHELXL97* (Sheldrick, 2008 ▶); molecular graphics: *SHELXTL* (Sheldrick, 2008 ▶); software used to prepare material for publication: *SHELXTL*.

## Supplementary Material

Crystal structure: contains datablock(s) I, global. DOI: 10.1107/S1600536811044291/hb6475sup1.cif

Structure factors: contains datablock(s) I. DOI: 10.1107/S1600536811044291/hb6475Isup2.hkl

Supplementary material file. DOI: 10.1107/S1600536811044291/hb6475Isup3.cml

Additional supplementary materials:  crystallographic information; 3D view; checkCIF report

## Figures and Tables

**Table 1:** Hydrogen-bond geometry (Å, °)

*D*—H⋯*A*	*D*—H	H⋯*A*	*D*⋯*A*	*D*—H⋯*A*
N2—H2⋯O2^i^	0.91 (1)	1.99 (1)	2.882 (2)	168 (4)

Symmetry code: (i) 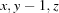.

**Fig. 1.**
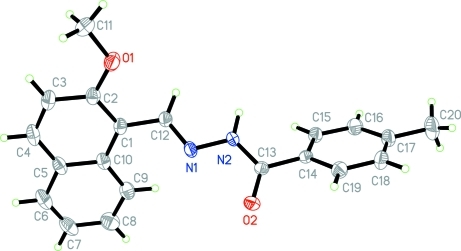
The molecular structure of the title compound, with displacement ellipsoids drawn at the 30% probability level.

**Fig. 2.**
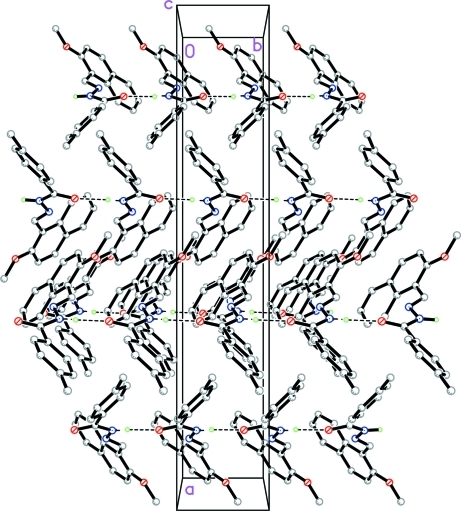
The crystal packing of the title compound, showing the N—H···O hydrogen-bonds (dashed lines) forming the chains propagating in the b axis direction. H-atoms not involved in the hydrogen bonding have been omitted for clarity.

## supplementary crystallographic information

### Comment

Benzohydrazide compounds are well known for their biological activities (El-Sayed *et al.*, 2011; Horiuchi *et al.*, 2009). In addition, benzohydrazide compounds have also been used as versatile ligands in coordination chemistry (El-Dissouky *et al.*, 2010, Zhang *et al.*, 2010). As a contribution to a structural study on hydrazone compounds, we present here the crystal structure of the title compound, that was obtained as the product of the reaction of 2-methoxy-1-naphthaldehyde with 4-methylbenzohydrazide in methanol.

In the title compound, Fig. 1, the mean planes of the naphthyl ring and the benzene ring form a dihedral angle of 91.5 (3)°. The bond distances and angles are within normal ranges (Allen *et al.*, 1987), and agree well with the corresponding bond distances and angles reported in closely related compounds (Suleiman Gwaram *et al.*, 2010; Liu *et al.*, 2011; Zhou *et al.*, 2011).

In the crystal, intermolecular N—H···O hydrogen bonds (Table 1) link the molecules to form chains which propagate along the *b* axis direction (Fig. 2).

### Experimental

To a methanol solution (20 ml) of 5-bromosalicylaldehyde (0.1 mmol, 20.1 mg) and 4-nitrobenzohydrazide (0.1 mmol, 18.1 mg), a few drops of acetic acid were added. The mixture was refluxed for 1 h and then cooled to room temperature. The white crystalline solid was collected by filtration, washed with cold methanol and dried in air. Colourless blocks were obtained by slow evaporation of a methanol solution of the product in air.

### Refinement

The NH H-atom was located in a difference Fourier map and was refined with a distance restraint, N—H = 0.90 (1) Å, and *U*_iso_(H) = 0.08 Å^2^. The OH and C-bound H atoms were positioned geometrically and refined using a riding model: O—H = 0.82 Å, C—H = 0.93 and 0.96 Å, for CH and CH_3_ H-atoms, respectively, with *U*_iso_(H) = k × *U*_eq_(O,*C*) where k = 1.5 for OH and CH_3_ H-atoms and k = 1.2 for all other H-atoms.

### Crystal data

**Table:**

C_20_H_18_N_2_O_2_	*F*(000) = 672
*M**_r_* = 318.36	*D*_x_ = 1.269 Mg m^−^^3^
Orthorhombic, *P**n**a*2_1_	Mo *K*α radiation, λ = 0.71073 Å
Hall symbol: P 2c -2n	Cell parameters from 3061 reflections
*a* = 26.738 (3) Å	θ = 2.7–24.6°
*b* = 4.893 (2) Å	µ = 0.08 mm^−^^1^
*c* = 12.735 (2) Å	*T* = 298 K
*V* = 1666.1 (8) Å^3^	Block, colorless
*Z* = 4	0.20 × 0.20 × 0.18 mm

### Data collection

**Table:**

Bruker SMART 1K CCD diffractometer	3622 independent reflections
Radiation source: fine-focus sealed tube	2579 reflections with *I* > 2σ(*I*)
graphite	*R*_int_ = 0.047
ω scan	θ_max_ = 27.0°, θ_min_ = 3.1°
Absorption correction: multi-scan (*SADABS*; Sheldrick, 1996)	*h* = −32→34
*T*_min_ = 0.984, *T*_max_ = 0.985	*k* = −6→6
12400 measured reflections	*l* = −16→16

### Refinement

**Table:**

Refinement on *F*^2^	Primary atom site location: structure-invariant direct methods
Least-squares matrix: full	Secondary atom site location: difference Fourier map
*R*[*F*^2^ > 2σ(*F*^2^)] = 0.043	Hydrogen site location: inferred from neighbouring sites
*wR*(*F*^2^) = 0.100	H atoms treated by a mixture of independent and constrained refinement
*S* = 1.03	*w* = 1/[σ^2^(*F*_o_^2^) + (0.0424*P*)^2^ + 0.1245*P*] where *P* = (*F*_o_^2^ + 2*F*_c_^2^)/3
3622 reflections	(Δ/σ)_max_ < 0.001
222 parameters	Δρ_max_ = 0.17 e Å^−^^3^
2 restraints	Δρ_min_ = −0.15 e Å^−^^3^

### Special details

**Table:**

Geometry. All e.s.d.'s (except the e.s.d. in the dihedral angle between two l.s. planes) are estimated using the full covariance matrix. The cell e.s.d.'s are taken into account individually in the estimation of e.s.d.'s in distances, angles and torsion angles; correlations between e.s.d.'s in cell parameters are only used when they are defined by crystal symmetry. An approximate (isotropic) treatment of cell e.s.d.'s is used for estimating e.s.d.'s involving l.s. planes.
Refinement. Refinement of *F*^2^ against ALL reflections. The weighted *R*-factor *wR* and goodness of fit *S* are based on *F*^2^, conventional *R*-factors *R* are based on *F*, with *F* set to zero for negative *F*^2^. The threshold expression of *F*^2^ > σ(*F*^2^) is used only for calculating *R*-factors(gt) *etc*. and is not relevant to the choice of reflections for refinement. *R*-factors based on *F*^2^ are statistically about twice as large as those based on *F*, and *R*- factors based on ALL data will be even larger.

### Fractional atomic coordinates and isotropic or equivalent isotropic displacement parameters (Å^2^)

**Table:**

	*x*	*y*	*z*	*U*_iso_*/*U*_eq_	
N1	0.39864 (7)	0.4057 (4)	0.22466 (14)	0.0406 (4)	
N2	0.37614 (7)	0.3174 (3)	0.31743 (15)	0.0377 (4)	
O1	0.50261 (6)	−0.0340 (4)	0.11479 (13)	0.0550 (4)	
O2	0.37197 (6)	0.7513 (3)	0.37919 (14)	0.0479 (4)	
C1	0.44133 (8)	0.2834 (5)	0.06656 (17)	0.0389 (5)	
C2	0.48465 (9)	0.1447 (5)	0.04022 (17)	0.0428 (6)	
C3	0.50956 (9)	0.1928 (6)	−0.05568 (19)	0.0531 (7)	
H3	0.5383	0.0953	−0.0726	0.064*	
C4	0.49103 (10)	0.3836 (5)	−0.1231 (2)	0.0553 (7)	
H4	0.5080	0.4173	−0.1856	0.066*	
C5	0.44714 (10)	0.5312 (5)	−0.10157 (17)	0.0470 (6)	
C6	0.42831 (12)	0.7288 (6)	−0.1722 (2)	0.0628 (8)	
H6	0.4459	0.7662	−0.2335	0.075*	
C7	0.38510 (12)	0.8652 (6)	−0.1524 (2)	0.0654 (8)	
H7	0.3734	0.9951	−0.1997	0.079*	
C8	0.35832 (11)	0.8088 (6)	−0.0604 (2)	0.0638 (7)	
H8	0.3284	0.8996	−0.0475	0.077*	
C9	0.37556 (10)	0.6224 (5)	0.01051 (19)	0.0519 (6)	
H9	0.3571	0.5889	0.0710	0.062*	
C10	0.42106 (9)	0.4781 (5)	−0.00566 (17)	0.0406 (5)	
C11	0.54715 (10)	−0.1814 (5)	0.0936 (2)	0.0588 (7)	
H11A	0.5419	−0.2991	0.0342	0.088*	
H11B	0.5559	−0.2895	0.1537	0.088*	
H11C	0.5737	−0.0555	0.0783	0.088*	
C12	0.41801 (8)	0.2176 (5)	0.16793 (16)	0.0392 (5)	
H12	0.4173	0.0374	0.1911	0.047*	
C13	0.36194 (8)	0.5068 (4)	0.38880 (16)	0.0353 (5)	
C14	0.33277 (8)	0.4017 (4)	0.47974 (16)	0.0339 (5)	
C15	0.29756 (8)	0.1938 (5)	0.46903 (18)	0.0431 (6)	
H15	0.2927	0.1121	0.4039	0.052*	
C16	0.26982 (9)	0.1080 (5)	0.5540 (2)	0.0520 (7)	
H16	0.2461	−0.0289	0.5448	0.062*	
C17	0.27637 (10)	0.2201 (5)	0.6521 (2)	0.0533 (6)	
C18	0.31194 (10)	0.4258 (5)	0.6632 (2)	0.0564 (7)	
H18	0.3174	0.5029	0.7289	0.068*	
C19	0.33931 (9)	0.5176 (5)	0.57820 (17)	0.0471 (6)	
H19	0.3623	0.6583	0.5871	0.056*	
C20	0.24619 (14)	0.1207 (8)	0.7453 (3)	0.0932 (12)	
H20A	0.2142	0.2093	0.7456	0.140*	
H20B	0.2637	0.1632	0.8090	0.140*	
H20C	0.2416	−0.0735	0.7402	0.140*	
H2	0.3739 (14)	0.134 (2)	0.327 (4)	0.140*	

### Atomic displacement parameters (Å^2^)

**Table:**

	*U*^11^	*U*^22^	*U*^33^	*U*^12^	*U*^13^	*U*^23^
N1	0.0471 (11)	0.0428 (11)	0.0319 (9)	−0.0044 (9)	0.0060 (9)	0.0064 (9)
N2	0.0451 (10)	0.0348 (10)	0.0333 (9)	−0.0005 (8)	0.0086 (8)	0.0070 (8)
O1	0.0488 (9)	0.0704 (11)	0.0460 (9)	0.0119 (8)	0.0065 (8)	0.0002 (9)
O2	0.0555 (10)	0.0324 (8)	0.0556 (10)	−0.0001 (7)	0.0122 (8)	0.0062 (8)
C1	0.0402 (13)	0.0453 (13)	0.0312 (11)	−0.0080 (11)	0.0031 (9)	−0.0036 (10)
C2	0.0435 (14)	0.0476 (14)	0.0373 (12)	−0.0080 (11)	0.0031 (11)	−0.0055 (11)
C3	0.0465 (14)	0.0647 (18)	0.0481 (15)	−0.0092 (12)	0.0131 (12)	−0.0086 (13)
C4	0.0606 (16)	0.0677 (16)	0.0376 (13)	−0.0278 (14)	0.0113 (13)	−0.0028 (14)
C5	0.0592 (16)	0.0480 (13)	0.0337 (12)	−0.0210 (12)	0.0000 (11)	0.0012 (11)
C6	0.082 (2)	0.0661 (18)	0.0405 (14)	−0.0274 (16)	−0.0026 (14)	0.0111 (14)
C7	0.088 (2)	0.0613 (17)	0.0475 (16)	−0.0176 (17)	−0.0151 (15)	0.0187 (14)
C8	0.0687 (18)	0.0642 (18)	0.0584 (17)	−0.0008 (15)	−0.0142 (15)	0.0072 (15)
C9	0.0550 (16)	0.0598 (16)	0.0409 (14)	−0.0032 (14)	−0.0017 (12)	0.0055 (13)
C10	0.0451 (13)	0.0432 (12)	0.0336 (11)	−0.0168 (11)	−0.0001 (10)	−0.0029 (11)
C11	0.0518 (15)	0.0591 (16)	0.0654 (17)	0.0086 (13)	0.0039 (13)	−0.0109 (14)
C12	0.0420 (12)	0.0425 (13)	0.0332 (11)	−0.0010 (10)	0.0029 (10)	0.0005 (11)
C13	0.0348 (11)	0.0361 (12)	0.0350 (12)	0.0067 (9)	−0.0028 (9)	0.0036 (10)
C14	0.0326 (11)	0.0335 (11)	0.0356 (11)	0.0062 (9)	0.0014 (9)	0.0009 (10)
C15	0.0413 (13)	0.0464 (14)	0.0415 (13)	0.0017 (11)	0.0048 (11)	0.0014 (11)
C16	0.0427 (15)	0.0519 (15)	0.0614 (18)	−0.0043 (12)	0.0133 (12)	0.0033 (14)
C17	0.0524 (15)	0.0604 (16)	0.0470 (15)	0.0088 (13)	0.0162 (12)	0.0103 (13)
C18	0.0699 (17)	0.0652 (17)	0.0341 (12)	0.0074 (14)	0.0070 (13)	−0.0037 (12)
C19	0.0527 (15)	0.0457 (13)	0.0428 (13)	−0.0022 (11)	0.0001 (12)	−0.0030 (12)
C20	0.100 (3)	0.114 (3)	0.066 (2)	0.006 (2)	0.0454 (18)	0.0203 (19)

### Geometric parameters (Å, °)

**Table:**

N1—C12	1.280 (3)	C8—H8	0.9300
N1—N2	1.394 (2)	C9—C10	1.422 (3)
N2—C13	1.352 (3)	C9—H9	0.9300
N2—H2	0.907 (10)	C11—H11A	0.9600
O1—C2	1.378 (3)	C11—H11B	0.9600
O1—C11	1.418 (3)	C11—H11C	0.9600
O2—C13	1.232 (2)	C12—H12	0.9300
C1—C2	1.384 (3)	C13—C14	1.488 (3)
C1—C10	1.431 (3)	C14—C19	1.387 (3)
C1—C12	1.469 (3)	C14—C15	1.393 (3)
C2—C3	1.411 (3)	C15—C16	1.378 (3)
C3—C4	1.362 (4)	C15—H15	0.9300
C3—H3	0.9300	C16—C17	1.376 (4)
C4—C5	1.405 (4)	C16—H16	0.9300
C4—H4	0.9300	C17—C18	1.392 (4)
C5—C6	1.413 (4)	C17—C20	1.515 (4)
C5—C10	1.430 (3)	C18—C19	1.381 (3)
C6—C7	1.358 (4)	C18—H18	0.9300
C6—H6	0.9300	C19—H19	0.9300
C7—C8	1.401 (4)	C20—H20A	0.9600
C7—H7	0.9300	C20—H20B	0.9600
C8—C9	1.364 (3)	C20—H20C	0.9600
			
C12—N1—N2	115.47 (17)	O1—C11—H11B	109.5
C13—N2—N1	118.57 (17)	H11A—C11—H11B	109.5
C13—N2—H2	124 (3)	O1—C11—H11C	109.5
N1—N2—H2	117 (3)	H11A—C11—H11C	109.5
C2—O1—C11	119.0 (2)	H11B—C11—H11C	109.5
C2—C1—C10	119.2 (2)	N1—C12—C1	120.7 (2)
C2—C1—C12	117.4 (2)	N1—C12—H12	119.7
C10—C1—C12	123.4 (2)	C1—C12—H12	119.7
O1—C2—C1	115.8 (2)	O2—C13—N2	122.5 (2)
O1—C2—C3	122.5 (2)	O2—C13—C14	121.80 (19)
C1—C2—C3	121.6 (2)	N2—C13—C14	115.69 (18)
C4—C3—C2	119.2 (2)	C19—C14—C15	118.19 (19)
C4—C3—H3	120.4	C19—C14—C13	119.75 (19)
C2—C3—H3	120.4	C15—C14—C13	122.04 (19)
C3—C4—C5	122.2 (2)	C16—C15—C14	120.6 (2)
C3—C4—H4	118.9	C16—C15—H15	119.7
C5—C4—H4	118.9	C14—C15—H15	119.7
C4—C5—C6	121.7 (2)	C17—C16—C15	121.6 (2)
C4—C5—C10	118.7 (2)	C17—C16—H16	119.2
C6—C5—C10	119.6 (2)	C15—C16—H16	119.2
C7—C6—C5	121.4 (3)	C16—C17—C18	117.8 (2)
C7—C6—H6	119.3	C16—C17—C20	121.0 (3)
C5—C6—H6	119.3	C18—C17—C20	121.1 (3)
C6—C7—C8	119.5 (3)	C19—C18—C17	121.2 (2)
C6—C7—H7	120.2	C19—C18—H18	119.4
C8—C7—H7	120.2	C17—C18—H18	119.4
C9—C8—C7	120.9 (3)	C18—C19—C14	120.6 (2)
C9—C8—H8	119.6	C18—C19—H19	119.7
C7—C8—H8	119.6	C14—C19—H19	119.7
C8—C9—C10	121.7 (2)	C17—C20—H20A	109.5
C8—C9—H9	119.2	C17—C20—H20B	109.5
C10—C9—H9	119.2	H20A—C20—H20B	109.5
C9—C10—C5	116.8 (2)	C17—C20—H20C	109.5
C9—C10—C1	124.2 (2)	H20A—C20—H20C	109.5
C5—C10—C1	119.0 (2)	H20B—C20—H20C	109.5
O1—C11—H11A	109.5		

### Hydrogen-bond geometry (Å, °)

**Table:**

*D*—H···*A*	*D*—H	H···*A*	*D*···*A*	*D*—H···*A*
N2—H2···O2^i^	0.91 (1)	1.99 (1)	2.882 (2)	168 (4)

Symmetry codes: (i) *x*, *y*−1, *z*.
